# The effects of the measures against COVID-19 pandemic on physical activity among school-aged children and adolescents (6–17 years) in 2020: A protocol for systematic review

**DOI:** 10.1371/journal.pone.0255520

**Published:** 2021-07-29

**Authors:** Donglin Hu, He Zhang, Yingshuang Sun, Yongqin Li

**Affiliations:** 1 School of Physical Education and Educational Science, Tianjin University of Sport, Tianjin, China; 2 Department of Physical Education, Nanjing Agricultural University, Nanjing, China; 3 Faculty of Health, Southern Cross University, Lismore, New South Wales, Australia; 4 Department of Graduate Studies, Shenyang Sport University, Shenyang, China; 5 College of Further Education, Nanjing Sport Institute, Nanjing, China; Xiamen University - Malaysia, MALAYSIA

## Abstract

**Background:**

The pandemic of coronavirus disease (COVID-19) has greatly changed people’s daily lives, forcing countries to take actions, such as school shutdown, lockdown, isolation, and social distancing measures. It remains unclear how the closures, cancellations, and restrictions of schools and courses as a response to the COVID-19 pandemic affect the engagement of school-aged children and adolescents in relation to physical activity (PA).

**Methods:**

The articles in the databases of EBSCO (including AMED, CINAHL Plus, Health Business, Health Source MEDLINE with Full Text, APA PsycArticles, APA PsycINFO, and SPORTDiscus) published during the period from 1 January 2020 to 31 December 2020 will be retrieved, and the data in the selected articles are extracted, including research methods, demographics, and key results. Search outcomes were reported according to the Preferred Reporting Items for Systematic Reviews and Meta-Analyses (PRISMA) guidelines. The Mixed Methods Appraisal Tool (MMAT) will be used to evaluate research quality. Two reviewers are responsible for completing the three tasks, namely selecting the articles that meet the inclusion criteria, extracting data in the articles selected, and evaluating their research quality. All findings, and especially primary outcomes will be summarized in a table format of findings. The results will provide a high-quality synthesis of current evidence for researchers in this subject area.

**Aim:**

The objective of this systematic review is to investigate the effects of the COVID-19 pandemic on PA in children and adolescents aged 6–17 years during 2020. 1). What impact has the COVID-19 pandemic had on PA levels in school-aged children and adolescents? 2). Investigating changes in the locations of school-aged children’s and adolescents’ PA between the pre-COVID-19 period (January 2020) and the COVID-19 period (December 2020).

**Results:**

We hope that this study will provide government authorities and health professionals with the necessary information in guiding actions and allocating resources, so that the situation of physical inactivity in school-aged children and adolescents during the COVID-19 pandemic can be improved, thereby enhancing their physical health.

**Protocol registration number:**

This review was submitted and registered under CRD42020225976 in PROSPERO.

## 1. Introduction

COVID-19 is a severe acute respiratory disease that involves multiple organ systems caused by the coronavirus. The disease is characterized by high morbidity and mortality [1]. On March 11, 2020, World Health Organization (WHO) declared the outbreak a global pandemic [2]. According to a report by WHO, as of December 31, 2020, there had been reported cases in more than 200 countries around the world, where the total number of global infection cases had exceeded 80 million and 1.7 million were dead, which were still rising [3]. The COVID-19 pandemic has greatly changed people’s daily lives, forcing countries to take actions, such as school shutdown, lockdown, isolation, and social distancing measures [4]. These efforts are expected to prevent the spread of the disease and reduce the pressure on the health care system. As a result, more than 2.6 billion people were forced to stay at home [5]. It remains unclear how the closures, cancellations, and restrictions of schools and courses as a response to the COVID-19 pandemic affect the engagement of children and adolescents in PA. School closures and the cancellation of physical education activity courses have prompted some coaches and fitness professionals to provide children with live lessons or recorded video lessons through social platforms or software like Zoom, YouTube, and Facebook. At the same time, a number of fitness apps have emerged [6]. What’s more, school closures have forced some students to spend more time at home without education. Due to various considerations and restrictions (e.g. family financial status, community safety, traffic safety, whether to have electronic devices), children do not have equal opportunities to do PA.

Physical activity refers to any bodily movement produced by skeletal muscles that requires energy expenditure [7, 8]. How to promote children and adolescents to do an appropriate level of PA has always been a major public health problem [8]. Consensus has been reached in the academic community that physical inactivity results in adverse health consequences [7–9]. Consequently, maintaining an appropriate level of PA for adolescents is of great benefits for their physical and mental health, such as promoting health in terms of the heart and lungs, muscles, bones, cardiovascular metabolism, body composition, and psychosocial health [10]. According to the recommendation given by the WHO, in order to achieve health benefits, children and adolescents should do 60 minutes or more of moderate-to-vigorous intensity PA each day [8]. However, more than 80% of children and adolescents in the world fall short of the recommended standard [8, 11]. Recently some studies [12–14] have suggested that COVID-19 pandemic may exacerbate physical inactivity and prolonged sedentary time in children, which may be attributed to the lack of equal opportunities to participate in PA and the unavailability of sports facilities caused by school closures. It has been shown that students have lower levels of PA and more sedentary time during weekends than in school [15, 16]. If school closures or physical education course cancellations as a result of the COVID-19 pandemic will cause physical inactivity in students just as they were on weekends, the longer the closure time, the more likely it will bring about severe consequences for children’s health. Therefore, this systematic review aims to 1). What impact has the COVID-19 pandemic had on PA levels in school-aged children and adolescents? 2). Investigating changes in the locations of school-aged children’s and adolescents’ PA between the pre-COVID-19 period (January 2020) and the COVID-19 period (December 2020). Given the fact that long-term physical inactivity is likely to increase children’s risk of obesity, diabetes, and other chronic diseases, the findings of this study regarding the impact of the COVID-19 pandemic on school-aged children’s and adolescents’ PA are expected to provide valuable information for government authorities and health professionals in guiding actions and allocating resources.

## 2. Methods and analysis

This review’s methods draw on the Preferred Reporting Items for Systematic Reviews and Meta-Analysis Protocols (PRISMA-P) guidelines (See S1 Checklist) [17]. Furthermore, the study protocol was registered on PROSPERO—international prospective register system of systematic reviews (Registration number: CRD42020225976). The development of the protocol began on 10 December 2020 and is registered on 21 December 2020. The study is expected to be completed 1 May 2021. In the final systematic review, we will include a PRISMA checklist. Since no raw data are collected in this manuscript, the approval of the ethics committee is not required.

### 2.1 Eligibility criteria

Prior to determining final eligibility criteria, we will perform a pretest. To this end, we will screen 50 titles/abstracts. The criteria will be adapted if needed following this pretest.

#### 2.1.1 Definition

- A school aged child: Primary and High School Student aged between 6 to 17 years.
- Adolescent: An Individual with Age of 6–17 years.

#### 2.1.2 Inclusion criteria

Inclusion criteria are:

- the research containing participants between 6 and 17 years of age (i.e. Primary school students & High school students);
- research participants were healthy children or adolescents (participants did not have any special diseases);
- research must report the PA locations and PA levels of children and adolescents before and during the COVID-19 pandemic.
- full-text available;
- written in English;
- published in scholarly (peer reviewed) journals.

#### 2.1.3 Exclusion criteria

Exclusion criteria are:

- studies focused on disabled and overweight populations only;
- books, book sections, dissertations, thesis, or conference abstracts.

A search of the literature was conducted on 15 Dec. 2020, through the following electronic databases: EBSCO (including AMED, CINAHL Plus, Health Business, Health Source MEDLINE with Full Text, APA PsycArticles, APA PsycINFO, and SPORTDiscus).

A Boolean search strategy will be used to identify articles that have a combination of the following keywords: (“COVID-19” or “2019-NCOV” or “COV-19”) AND (“physical activity” or “exercise” or “fitness” or “physical exercise” or “sport”) AND (children or adolescents or youth or child or teenager or teens). Search outcomes will be reported according to the PRISMA guidelines.

The literature published between 2020 (January) to 2020 (December) will be retrieved.

### 2.2 Data management

The recorded data will be systematically assembled into groups linked to database origin, and imported to EndNote X9 desktop version 9.3.3 (Thomson Research Soft Corp., USA).

### 2.3 Study selection process

Two reviewers (DLH and YSS) will search the databases and assess the articles’ titles and abstracts separately to determine the initial inclusions. If discrepancies will be found and can not be resolved between the two researchers, a third researcher (YQL) will be engaged to finalize the assessment.

### 2.4. Data collection process and data items

The study selection process is based on the PRISMA flow diagram (See S1 File) [17]. Two researchers will search the databases and assess the title and abstract of the articles separately to determine the initial inclusions. The full texts will be assessed against the inclusion and exclusion criteria, to finalize the articles eligible for inclusion in the review. If necessary, authors will be contacted to provide additional information.

The information to be extracted from the full text covers methodological, demographic, and outcome data, specifically including:

Reported participants’ characteristics (number of participants, participant age range, gender, nationality, school grade).
The definition of PA.
The location of studies.
Research methods.
Severity of restricted internal movement by country (i.e. localized and national lockdown, campus closure, etc.).

Collection methods to assess PA (i.e. questionnaires / interview).
Instruments used (i.e. accelerometers and heart rate monitors, self-report).
Findings/outcomes (i.e. the metabolic equivalents, the MET-minutes).

Next, both reviewers will extract findings related to PA levels in children or adolescents from “pre-pandemic” to “during the pandemic”.

### 2.5 Assessment of study quality/risk of bias

Two reviewers will independently rate the quality of the included studies using the Mixed Methods Appraisal Tool (MMAT) for observational studies [18]. Two reviewers will use a checklist considering risk of bias resulting from study design, randomization, allocation concealment and blinding, selection, attrition, assessment, intervention fidelity, analysis and handling of confounding. Any discrepancies will be resolved by a third reviewer. The quality of the articles that meet the inclusion criteria is strictly assessed by the MMAT about whether the research method is reasonable, whether the research design is biased, how the research is conducted, and what methods are used for data analysis. The MMAT is designed for the appraisal of mixed studies using qualitative, quantitative, or mixed methods. For different types of research, the MMAT contains two screening questions; for each of the two possible research design types, five questions are developed to assess research quality (See S2 File).

### 2.6 Data synthesis

A minimum of two studies that agreed in the results will be necessary to consider the data. All findings, and especially primary outcomes, (PA levels, PA time, locations of child’s PA, and demographics) will be summarized in a table format of findings. The extracted details will include the metabolic equivalents (METs), the MET-minutes (MET-mins), measurement instruments used for location assessment (e.g., Global Positioning Systems (GPS), Geographic Information Systems (GIS) or self-reported tools), description of locations (e.g., home, neighborhood, school, recreational sites). Using the information gathered from included studies a narrative synthesis of the literature will be created. Descriptive statistics will be used to report characteristics of included studies.

A meta-analysis will not be conducted due to the variation in the impact of the COVID-19 pandemic, the approaches taken by governments across affected countries, the variability in the assessment of PA and the quality of available literature.

### 2.7 Patient and public involvement statement

The present review protocol did not involve individual patients or public agencies.

## 3. Discussion

While some recent studies [6, 19–21] have reported the changes in children and adolescents’ PA during the COVID-19 period, no study has systematically synthesized the differences in school-aged children and adolescents’ PA during the COVID-19 period, and the factors associated with it. Therefore, our aim is to close this gap in knowledge. In addition, the MMAT is to be used to evaluate research quality.

### 3.1 Strengths and limitations

To the best of our knowledge, this is the first study protocol that systematically summarizes the impact of the COVID-19 pandemic on school-aged children and adolescents with PA. The main advantage lies in that literature selection, data extraction, and literature quality appraisal will be performed by two reviewers. Only peer-reviewed literature will be selected, thereby ensuring the quality of the selected literature. It can be considered as an advantage.

This study also has some limitations to be considered. First of all, some important articles (e.g. grey literature and non-English literature) may be excluded. Although we retrieve some major electronic databases, such as MEDLINE Database, the possibility cannot be ruled out that there are more important articles in other databases. Another limitation of this study is that the COVID-19 pandemic has caused varying impacts on different countries that have adopted miscellaneous response measures. Therefore, our findings may be inapplicable to the countries that are not included in the scope of this study. In the end, consideration should be paid to the fact that until now in 2021, the pandemic has not yet come to an end, so that many of its effects remain unclear and have always been a matter of concern to the scientific community. Therefore, it can be expected that more research results will be published. Finally, it is important to consider that new studies are constantly ongoing because so many COVID-19 impacts remain unknown and the pandemic continues to be a matter of concern for both the public and the scientific community.

## 4. Conclusions

Quality findings in a systematic review study depends on inclusion and searching criteria to obtain a quality data and how the data is processed and interpreted. Our systematic review may identify possible gaps in knowledge, such as existing studies may mainly rely on self-reported questionnaires [22–25], which could emphasize the need for future studies based on objective assessments (e.g. accelerometers and heart rate monitors or other valid methods). Furthermore, this study will examine the effects of the COVID-19 pandemic on PA levels in school-aged children and adolescents. The results are expected to provide government authorities and health professionals with the necessary information in guiding actions and allocating resources. This aims to improve the situation of physical inactivity in school-aged children and adolescents during the COVID-19 pandemic, thus enhancing their physical health.

## Supporting information

S1 Checklist PRISMA-P 2015 checklist: Recommended items to address in a systematic review protocol. (DOC)

S1 File The PRISMA flow diagram. (TIF)

S2 File The Mixed Methods Appraisal Tool (MMAT). (ZIP)

S3 File Timetable. (TIF)
