# Supplementary figures and images for: The effects of the measures against COVID-19 pandemic on physical activity among school-aged children and adolescents (6–17 years) in 2020: A protocol for systematic review
Source: PLoS One. 2021 Jul 29;16(7):e0255520. doi: 10.1371/journal.pone.0255520 (PMC8320922; doi:10.1371/journal.pone.0255520)

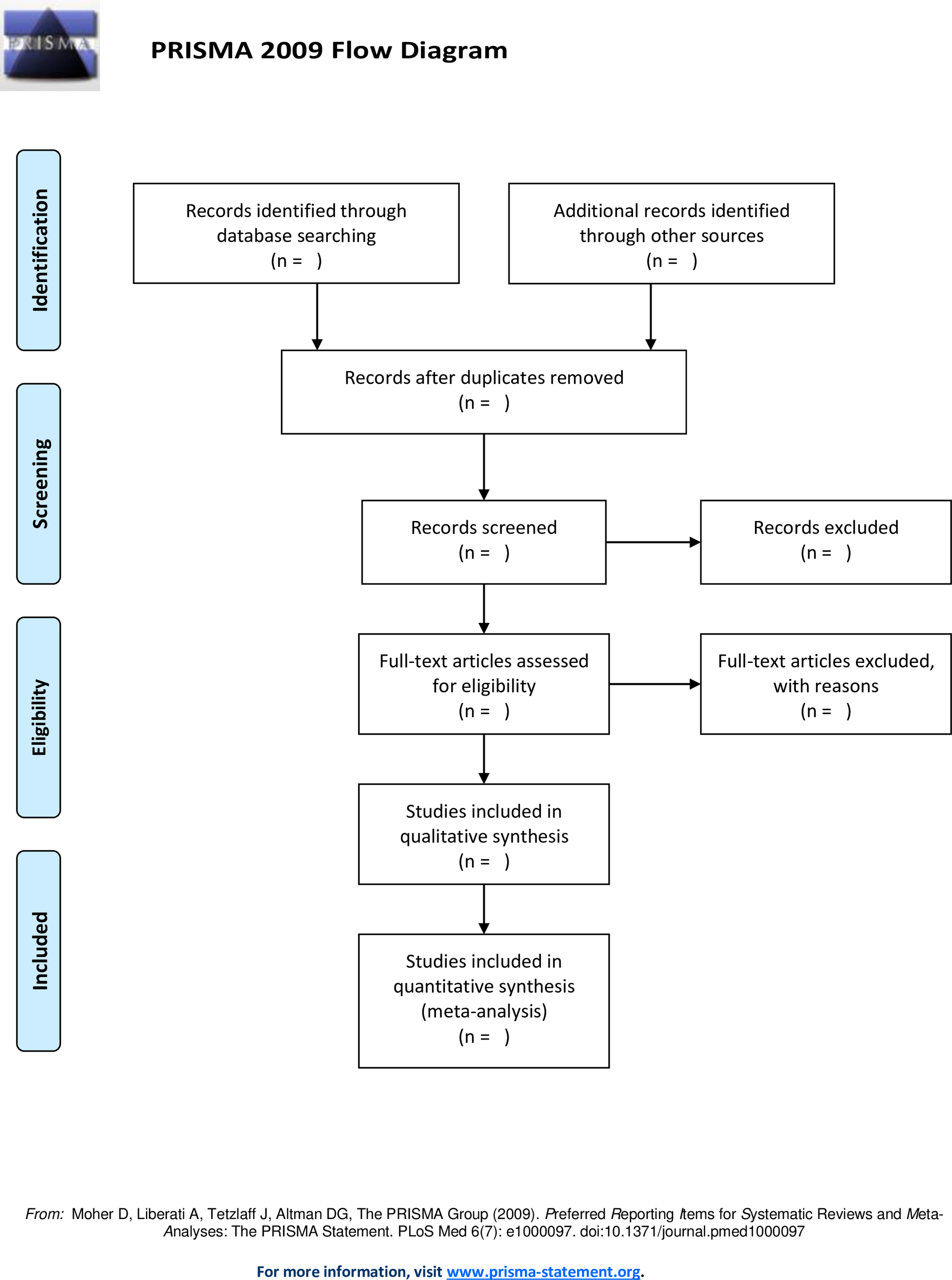

Supplement: S1 File — (TIF) [file pone.0255520.s002.tif]

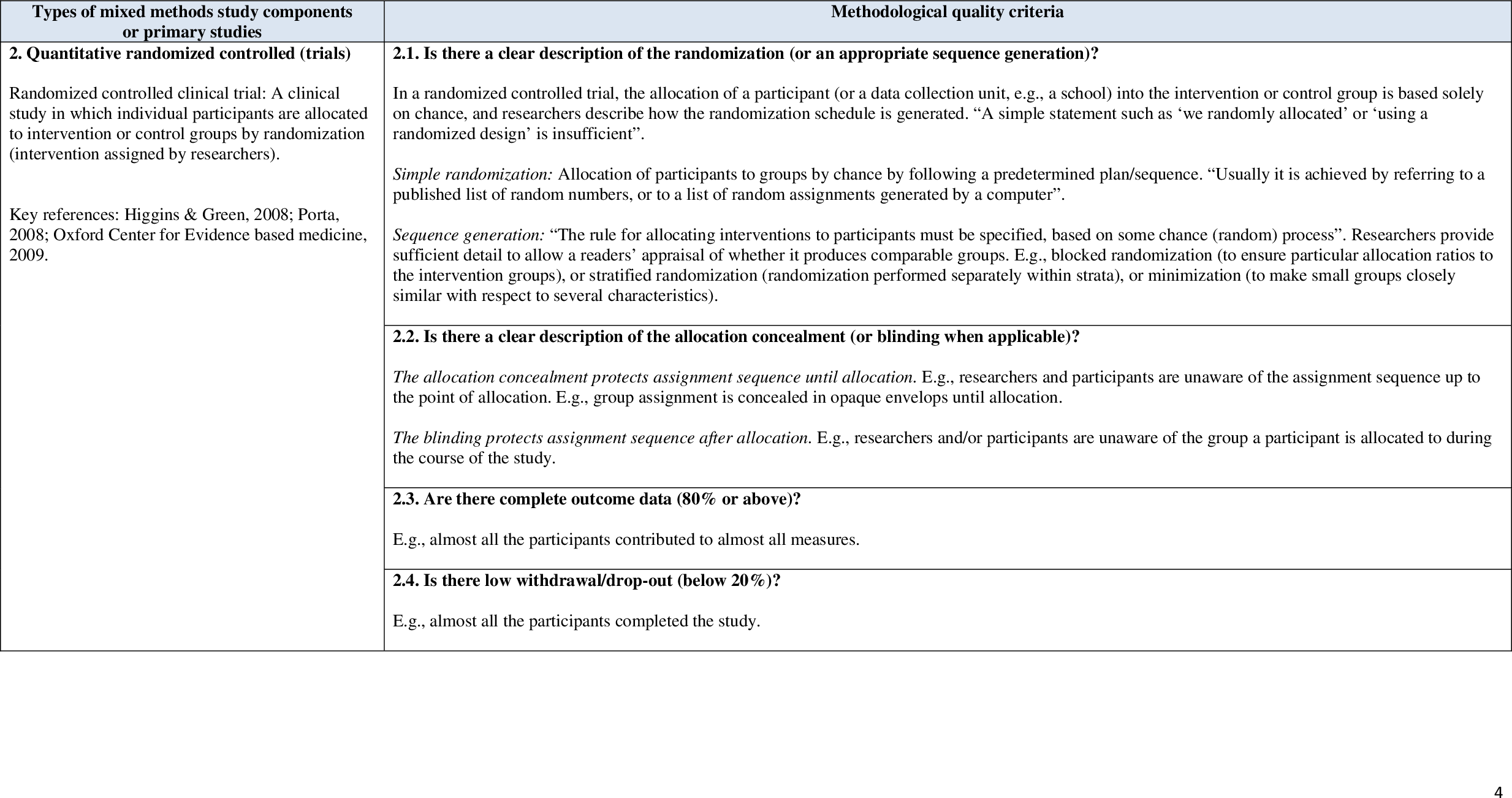

Supplement: S2 File — (ZIP) [file pone.0255520.s003.zip › S3.2.tif]

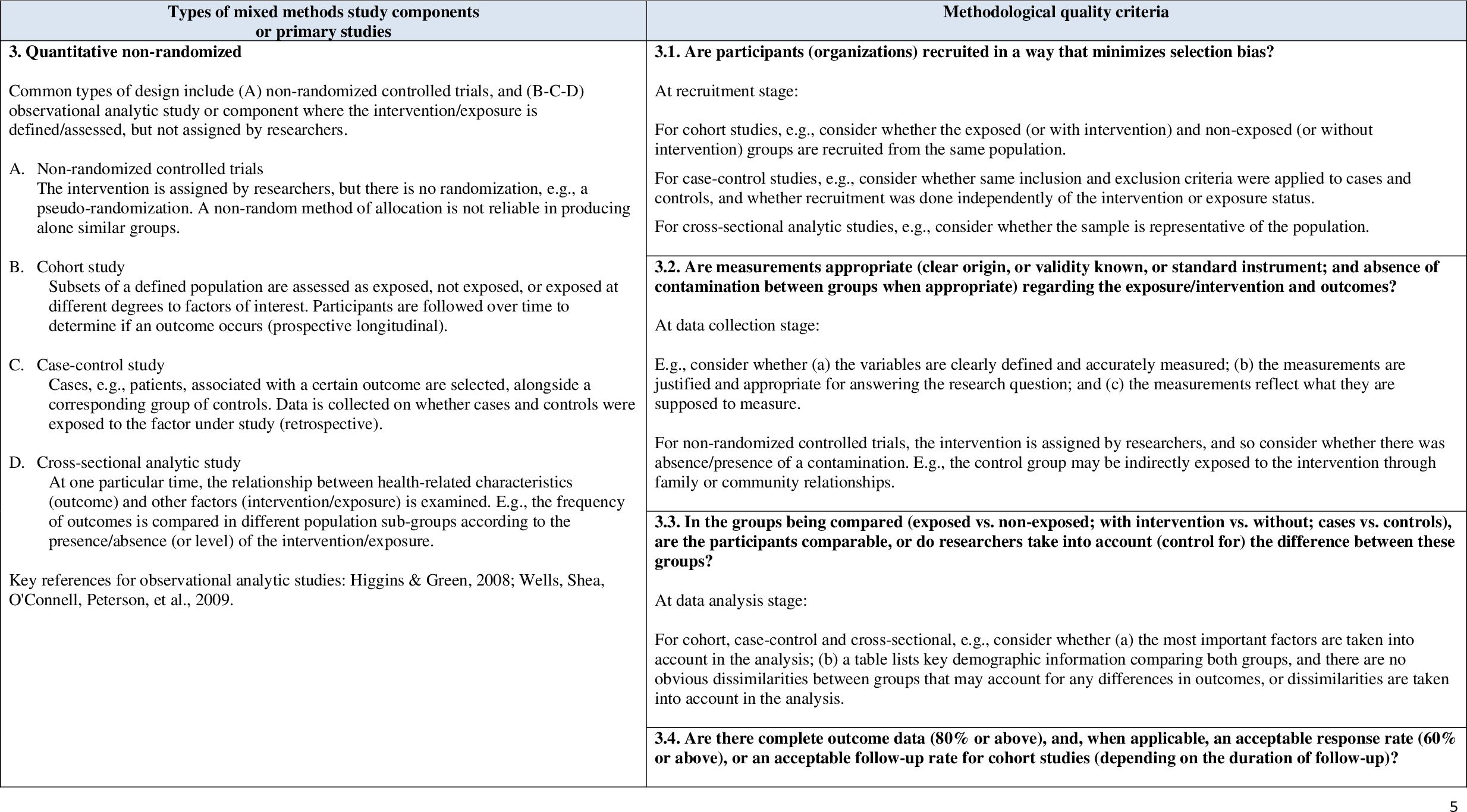

Supplement: S2 File — (ZIP) [file pone.0255520.s003.zip › S3.3.tif]

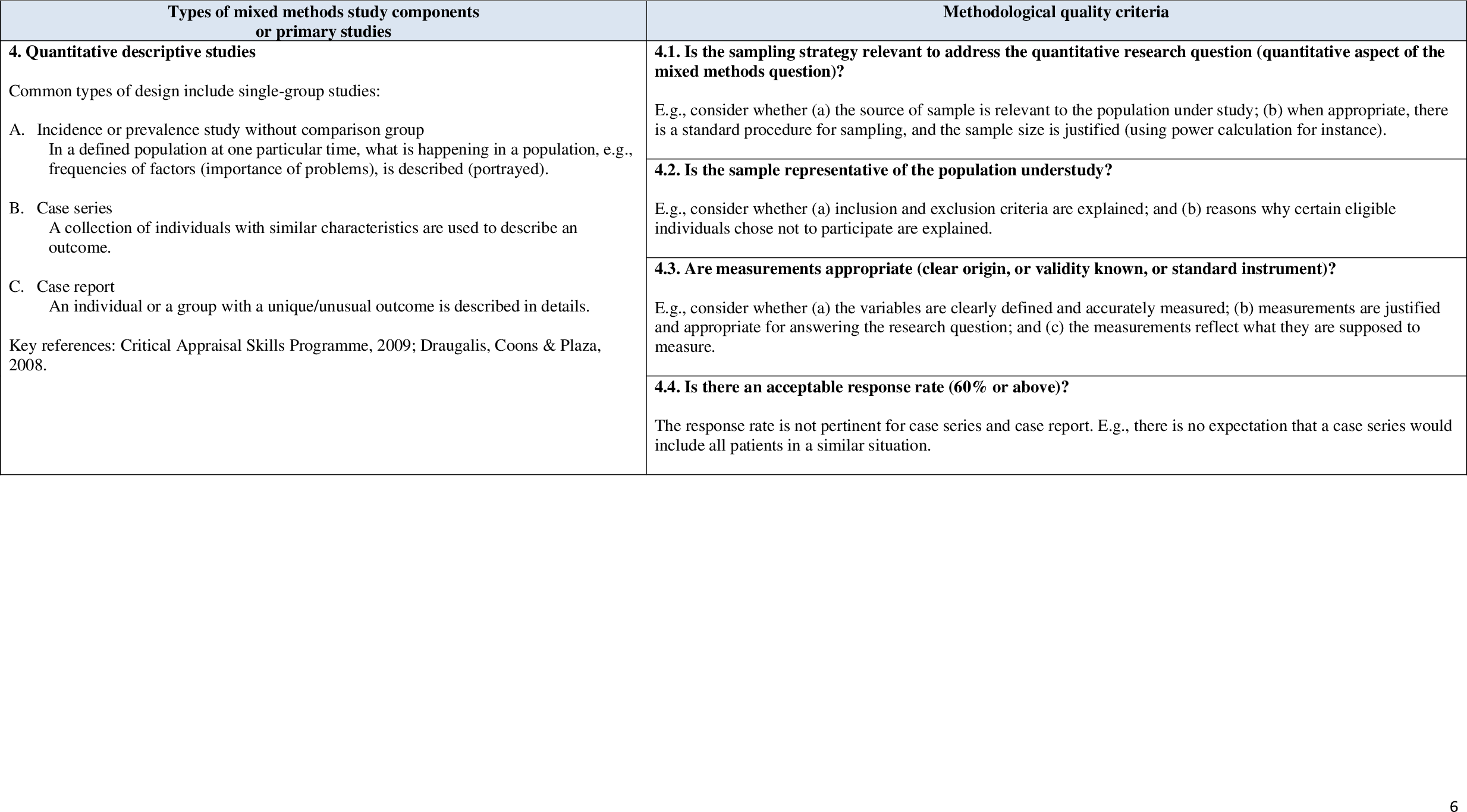

Supplement: S2 File — (ZIP) [file pone.0255520.s003.zip › S3.4.tif]

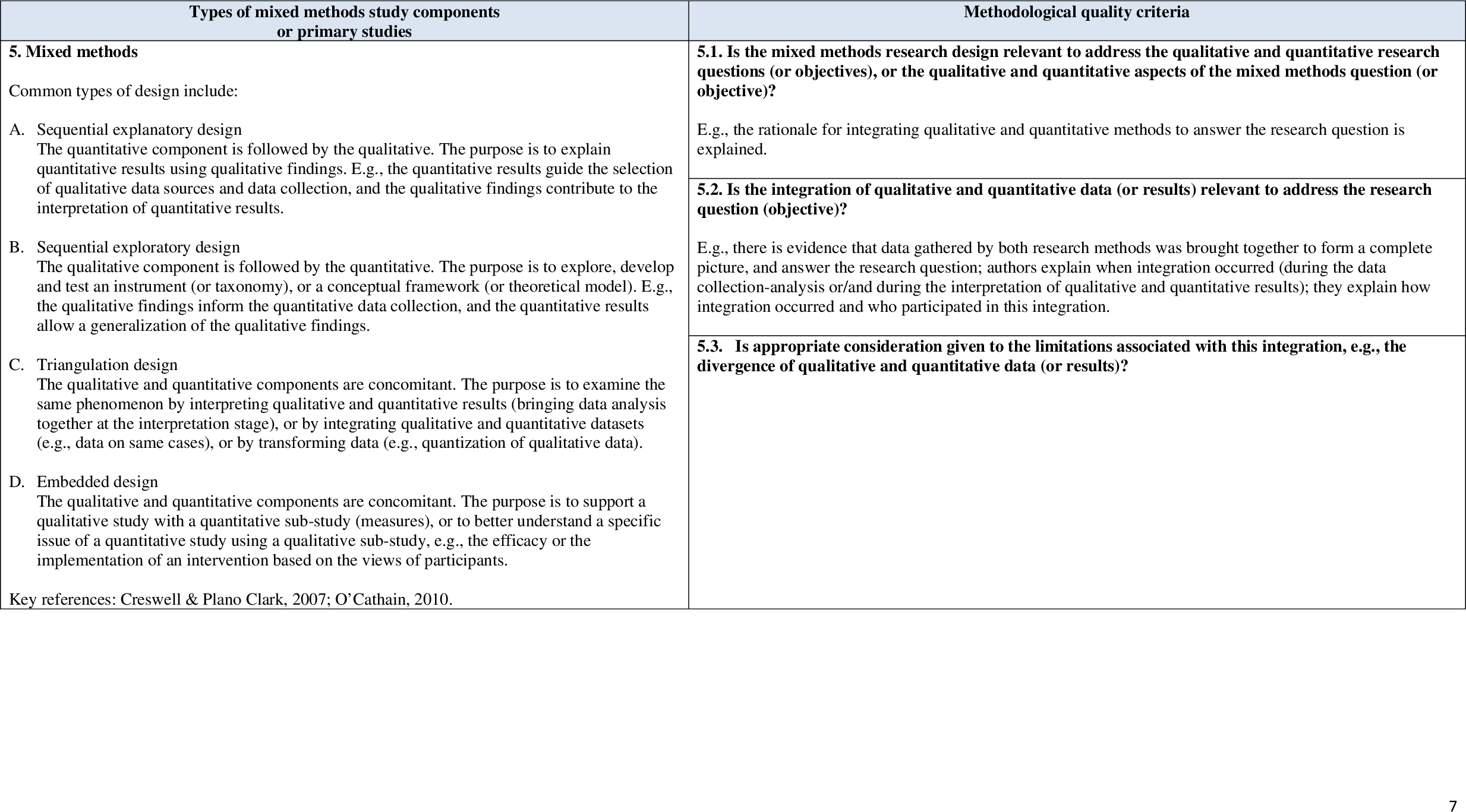

Supplement: S2 File — (ZIP) [file pone.0255520.s003.zip › S3.5.tif]

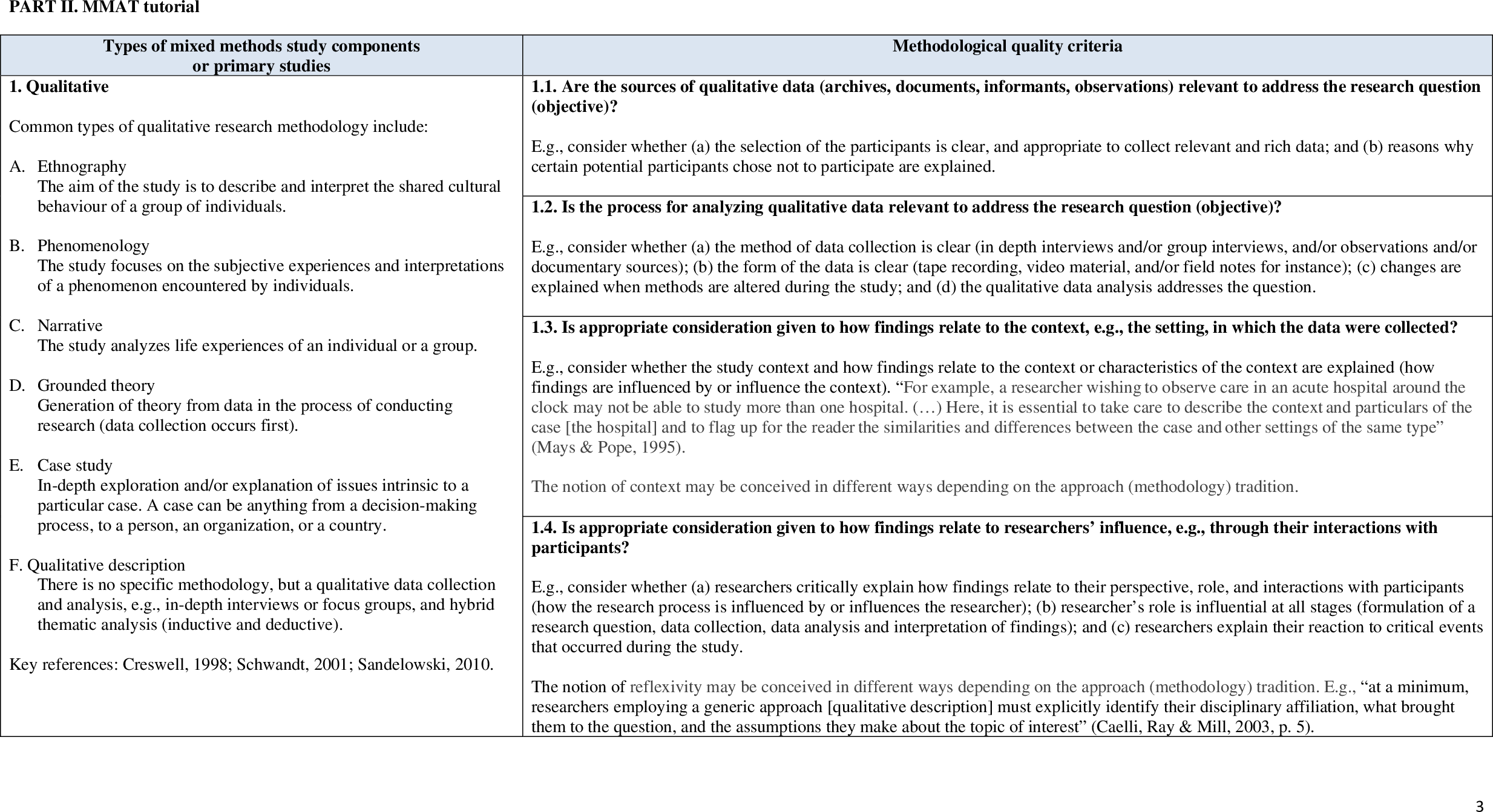

Supplement: S2 File — (ZIP) [file pone.0255520.s003.zip › S3.1.tif]

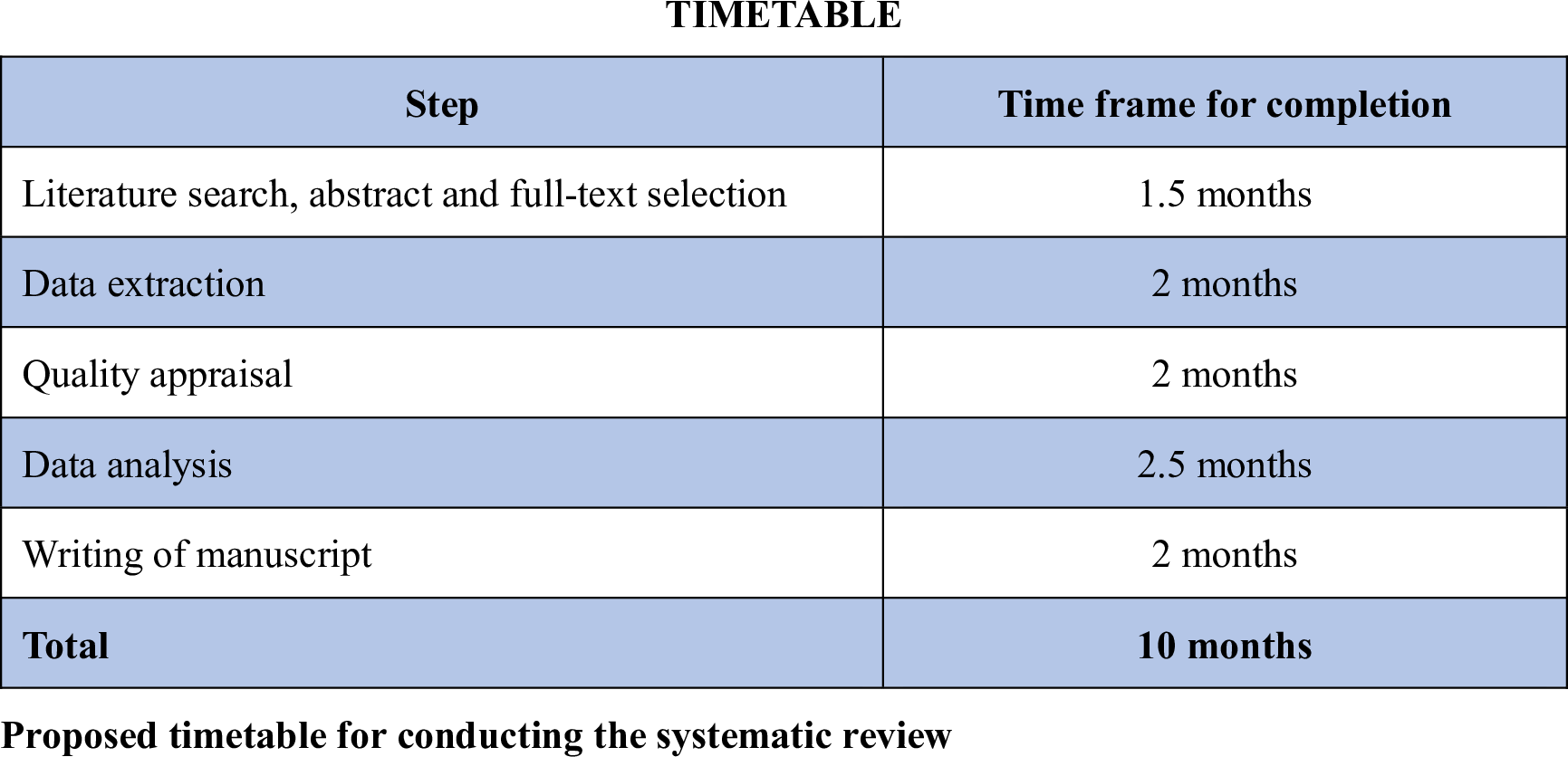

Supplement: S3 File — (TIF) [file pone.0255520.s004.tif]
